# 7,8-Dimethyl-11*H*-indeno­[1,2-*b*]quinoxalin-11-one

**DOI:** 10.1107/S2414314621000183

**Published:** 2021-01-08

**Authors:** Lin Chen, Jin Hu, Hong-Shun Sun

**Affiliations:** a Targeted MRI Contrast Agents Laboratory of Jiangsu Province, Nanjing Polytechnic Institute, Nanjing 210048, People’s Republic of China; b School of Biology and Environment, Nanjing Polytechnic Institute, Nanjing 210048, People’s Republic of China; Zhejiang University (Yuquan Campus), China

**Keywords:** crystal structure, indene, quinoxaline, indeno­[1,2-*b*]quinoxaline

## Abstract

In the title compound, C_17_H_12_N_2_O, the mean planes of the indene ring and quinoxaline system (r.m.s. deviations = 0.0131 and 0.0082 Å) are approximately parallel to one another, making a dihedral angle of 1.2 (5)°. This means that the indeno­[1,2-*b*]quinoxaline ring is almost in the same plane (r.m.s. deviation = 0.0181 Å).

## Abstract 2

In the title compound, the mean planes of the indene ring and quinoxaline system are approximately parallel to one another, making a dihedral angle of 1.2 (5)°. The indeno­[1,2-*b*]quinoxaline ring is almost in the same plane.

## Graphical abstract

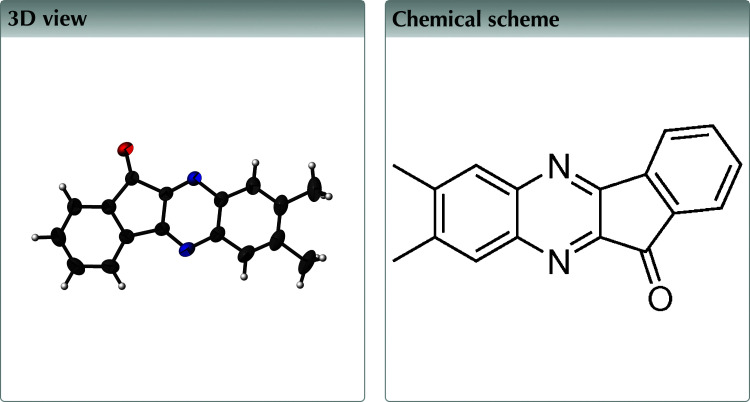

## Structure description

Quinoxaline based N-heteroacenes show a narrow band-gap, high thermal stability and aligned film morphology and can be applied as the hole-transport layers in quantum dot light-emitting diodes (QLEDs) (Bai *et al.*, 2015 ▸). As part of our work in this area, we now report the synthesis and crystal structure of the title indeno­[1,2-*b*]quinoxaline derivative.

**Figure 1:**
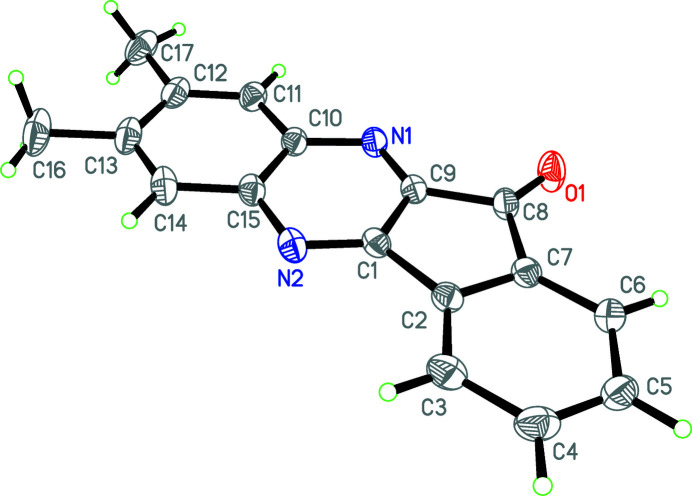
The mol­ecular structure of the title mol­ecule with the atom-labelling scheme. Displacement ellipsoids are drawn at the 30% probability level.

**Figure 2:**
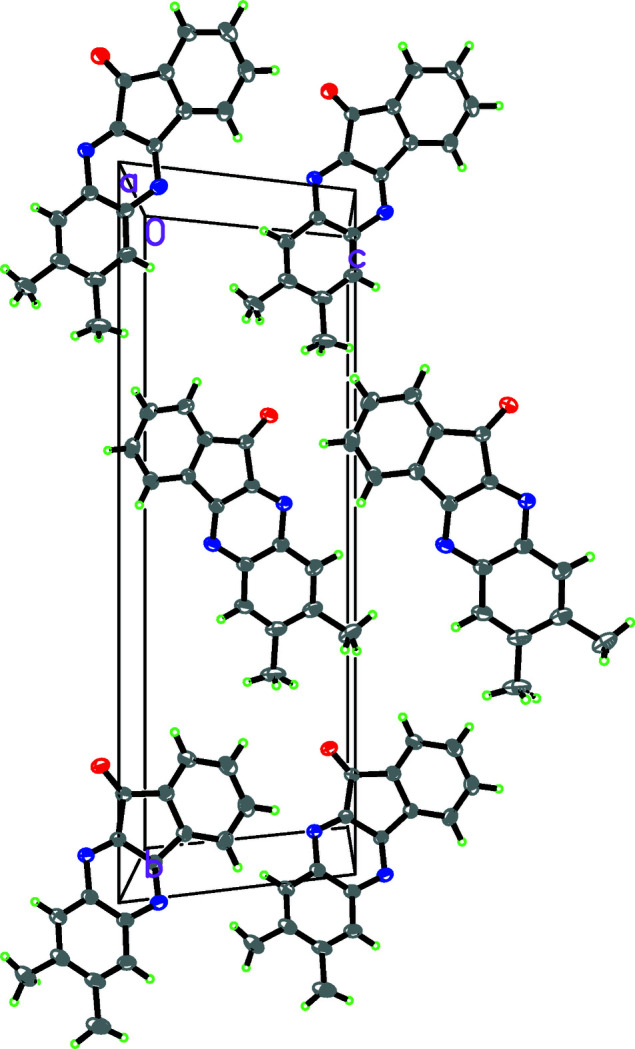
A packing diagram of the title compound.

The mol­ecular structure of the title compound is shown in Fig. 1 ▸. The indene ring and quinoxaline system are nearly parallel to one another [dihedral angle = 1.2 (5)°]. This means that the indeno­[1,2-*b*]quinoxaline ring (N1–N2/C1–C15) is almost in the same plane (r.m.s. deviation = 0.0181 Å), which contains the two methyl groups. The packing is shown in Fig. 2 ▸.

One similar structure has been reported previously (11,11-diphenyl-11*H*-indeno­[1,2-*b*]quinoxaline; Chen *et al.*, 2020 ▸). In that structure, the indeno­[1,2-*b*]quinoxaline ring (r.m.s. deviations = 0.1197 Å) is twisted with respect to the two benzene ring systems by 70.0 (4) and 67.6 (3)°, respectively.

## Synthesis and crystallization

A mixture of 1*H*-indene-1,2,3-trione (3.20 g, 20 mmol) and 4,5-di­methyl­benzene-1,2-di­amine (2.72 g, 20 mmol) in ethanol (100 ml) was heated to reflux under stirring for 5 h. 7,8-Dimethyl-11*H*-indeno­[1,2-*b*]quinoxalin-11-one was obtained as a yellow powder by filtering after cooling, yield 82%. Single crystals of the title compound suitable for X-ray analysis were obtained by slow evaporation of a methanol solution.

## Refinement

Crystal data, data collection and structure refinement details are summarized in Table 1 ▸.

## Supplementary Material

Crystal structure: contains datablock(s) I. DOI: 10.1107/S2414314621000183/xu4042sup1.cif

Structure factors: contains datablock(s) I. DOI: 10.1107/S2414314621000183/xu4042Isup2.hkl

Supporting information file. DOI: 10.1107/S2414314621000183/xu4042Isup3.cml

CCDC reference: 2054191

Additional supporting information:  crystallographic information; 3D view; checkCIF report

## Figures and Tables

**Table 1:** Experimental details

Crystal data	
Chemical formula	C_17_H_12_N_2_O
*M*_r_	260.29
Crystal system, space group	Monoclinic, *P*2_1_/*n*
Temperature (K)	282
*a*, *b*, *c* (Å)	7.4548 (9), 22.976 (3), 8.3149 (10)
β (°)	115.866 (3)
*V* (Å^3^)	1281.5 (3)
*Z*	4
Radiation type	Mo *K*α
μ (mm^−1^)	0.09
Crystal size (mm)	0.45 × 0.3 × 0.22

Data collection	
Diffractometer	Bruker SMART CCD area detector
Absorption correction	Multi-scan (*SADABS*; Bruker, 2014 ▸)
*T*_min_, *T*_max_	0.606, 0.746
No. of measured, independent and observed [*I* > 2σ(*I*)] reflections	10114, 3134, 2437
*R*_int_	0.027
(sin θ/λ)_max_ (Å^−1^)	0.668

Refinement	
*R*[*F* ^2^ > 2σ(*F* ^2^)], *wR*(*F* ^2^), *S*	0.060, 0.153, 1.05
No. of reflections	3134
No. of parameters	183
H-atom treatment	H-atom parameters constrained
Δρ_max_, Δρ_min_ (e Å^−3^)	0.27, −0.23

Computer programs: *SMART* and *SAINT* (Bruker, 2014 ▸), *SHELXT* (Sheldrick, 2015*a* ▸), *SHELXL* (Sheldrick, 2015*b* ▸) and *OLEX2* (Dolomanov *et al.*, 2009 ▸).

## full crystallographic data

### Crystal data

**Table:**

C_17_H_12_N_2_O	*F*(000) = 544
*M**_r_* = 260.29	*D*_x_ = 1.349 Mg m^−^^3^
Monoclinic, *P*2_1_/*n*	Mo *K*α radiation, λ = 0.71073 Å
*a* = 7.4548 (9) Å	Cell parameters from 5967 reflections
*b* = 22.976 (3) Å	θ = 2.7–28.4°
*c* = 8.3149 (10) Å	µ = 0.09 mm^−^^1^
β = 115.866 (3)°	*T* = 282 K
*V* = 1281.5 (3) Å^3^	Block, colorless
*Z* = 4	0.45 × 0.3 × 0.22 mm

### Data collection

**Table:**

Bruker SMART CCD 6000 area detector diffractometer	3134 independent reflections
Radiation source: sealed X-ray tube	2437 reflections with *I* > 2σ(*I*)
Graphite monochromator	*R*_int_ = 0.027
Detector resolution: 5.6 pixels mm^-1^	θ_max_ = 28.4°, θ_min_ = 3.1°
ω scans	*h* = −9→9
Absorption correction: multi-scan (SADABS; Bruker, 2014)	*k* = −30→30
*T*_min_ = 0.606, *T*_max_ = 0.746	*l* = −11→5
10114 measured reflections	

### Refinement

**Table:**

Refinement on *F*^2^	Primary atom site location: structure-invariant direct methods
Least-squares matrix: full	Hydrogen site location: inferred from neighbouring sites
*R*[*F*^2^ > 2σ(*F*^2^)] = 0.060	H-atom parameters constrained
*wR*(*F*^2^) = 0.153	*w* = 1/[σ^2^(*F*_o_^2^) + (0.0487*P*)^2^ + 0.7679*P*] where *P* = (*F*_o_^2^ + 2*F*_c_^2^)/3
*S* = 1.05	(Δ/σ)_max_ = 0.001
3134 reflections	Δρ_max_ = 0.27 e Å^−^^3^
183 parameters	Δρ_min_ = −0.22 e Å^−^^3^
0 restraints	

### Special details

**Table:**

Geometry. All esds (except the esd in the dihedral angle between two l.s. planes) are estimated using the full covariance matrix. The cell esds are taken into account individually in the estimation of esds in distances, angles and torsion angles; correlations between esds in cell parameters are only used when they are defined by crystal symmetry. An approximate (isotropic) treatment of cell esds is used for estimating esds involving l.s. planes.

### Fractional atomic coordinates and isotropic or equivalent isotropic displacement parameters (Å^2^)

**Table:**

	*x*	*y*	*z*	*U*_iso_*/*U*_eq_	
O1	0.1899 (2)	0.67104 (6)	0.38760 (19)	0.0613 (4)	
N1	0.1135 (2)	0.54175 (6)	0.3195 (2)	0.0400 (4)	
N2	0.3665 (2)	0.48258 (6)	0.6458 (2)	0.0417 (4)	
C1	0.3614 (2)	0.53906 (7)	0.6270 (2)	0.0348 (4)	
C2	0.4780 (2)	0.58312 (7)	0.7599 (2)	0.0366 (4)	
C3	0.6183 (3)	0.57743 (9)	0.9349 (3)	0.0483 (5)	
H3	0.6505	0.5411	0.9892	0.058*	
C4	0.7104 (3)	0.62748 (10)	1.0279 (3)	0.0542 (5)	
H4	0.8068	0.6244	1.1455	0.065*	
C5	0.6616 (3)	0.68157 (9)	0.9493 (3)	0.0517 (5)	
H5	0.7257	0.7144	1.0146	0.062*	
C6	0.5187 (3)	0.68793 (8)	0.7744 (3)	0.0463 (5)	
H6	0.4851	0.7245	0.7217	0.056*	
C7	0.4275 (3)	0.63822 (8)	0.6807 (2)	0.0381 (4)	
C8	0.2716 (3)	0.63268 (7)	0.4928 (2)	0.0400 (4)	
C9	0.2358 (2)	0.56799 (7)	0.4648 (2)	0.0362 (4)	
C10	0.1132 (3)	0.48201 (7)	0.3342 (2)	0.0381 (4)	
C11	−0.0140 (3)	0.44895 (8)	0.1861 (3)	0.0448 (4)	
H11	−0.0943	0.4680	0.0802	0.054*	
C12	−0.0229 (3)	0.38935 (8)	0.1933 (3)	0.0490 (5)	
C13	0.1007 (3)	0.36040 (8)	0.3545 (3)	0.0528 (5)	
C14	0.2283 (3)	0.39190 (8)	0.4996 (3)	0.0500 (5)	
H14	0.3106	0.3723	0.6037	0.060*	
C15	0.2382 (3)	0.45313 (7)	0.4951 (2)	0.0389 (4)	
C16	0.0931 (4)	0.29501 (9)	0.3674 (4)	0.0773 (8)	
H16A	0.1901	0.2825	0.4830	0.116*	
H16B	0.1219	0.2775	0.2765	0.116*	
H16C	−0.0375	0.2834	0.3511	0.116*	
C17	−0.1623 (4)	0.35635 (10)	0.0302 (4)	0.0665 (7)	
H17A	−0.2286	0.3831	−0.0665	0.100*	
H17B	−0.2595	0.3362	0.0560	0.100*	
H17C	−0.0878	0.3287	−0.0030	0.100*	

### Atomic displacement parameters (Å^2^)

**Table:**

	*U*^11^	*U*^22^	*U*^33^	*U*^12^	*U*^13^	*U*^23^
O1	0.0802 (11)	0.0335 (7)	0.0499 (8)	0.0066 (7)	0.0095 (7)	0.0056 (6)
N1	0.0444 (8)	0.0322 (7)	0.0406 (8)	0.0024 (6)	0.0159 (7)	0.0001 (6)
N2	0.0491 (9)	0.0341 (7)	0.0459 (9)	0.0071 (6)	0.0245 (7)	0.0067 (6)
C1	0.0365 (8)	0.0343 (8)	0.0375 (9)	0.0055 (6)	0.0197 (7)	0.0032 (7)
C2	0.0345 (8)	0.0395 (9)	0.0381 (9)	0.0041 (7)	0.0179 (7)	−0.0004 (7)
C3	0.0451 (10)	0.0541 (11)	0.0408 (10)	0.0099 (8)	0.0143 (8)	0.0063 (8)
C4	0.0406 (10)	0.0733 (14)	0.0405 (10)	0.0042 (9)	0.0102 (8)	−0.0085 (10)
C5	0.0409 (10)	0.0557 (12)	0.0533 (12)	−0.0030 (8)	0.0157 (9)	−0.0163 (9)
C6	0.0435 (10)	0.0409 (10)	0.0517 (11)	0.0003 (8)	0.0182 (9)	−0.0077 (8)
C7	0.0371 (9)	0.0388 (9)	0.0399 (9)	0.0026 (7)	0.0181 (8)	−0.0024 (7)
C8	0.0463 (10)	0.0313 (8)	0.0395 (9)	0.0029 (7)	0.0161 (8)	−0.0019 (7)
C9	0.0386 (8)	0.0317 (8)	0.0389 (9)	0.0030 (6)	0.0175 (7)	0.0015 (7)
C10	0.0419 (9)	0.0337 (8)	0.0457 (10)	−0.0001 (7)	0.0257 (8)	−0.0023 (7)
C11	0.0485 (10)	0.0413 (10)	0.0499 (11)	−0.0045 (8)	0.0264 (9)	−0.0078 (8)
C12	0.0565 (11)	0.0417 (10)	0.0663 (13)	−0.0115 (8)	0.0429 (11)	−0.0148 (9)
C13	0.0715 (13)	0.0330 (9)	0.0784 (15)	−0.0068 (9)	0.0554 (12)	−0.0073 (9)
C14	0.0690 (13)	0.0325 (9)	0.0617 (12)	0.0054 (8)	0.0408 (11)	0.0060 (8)
C15	0.0467 (9)	0.0320 (8)	0.0488 (10)	0.0026 (7)	0.0308 (8)	0.0015 (7)
C16	0.114 (2)	0.0324 (10)	0.112 (2)	−0.0109 (12)	0.0750 (19)	−0.0078 (12)
C17	0.0766 (15)	0.0572 (13)	0.0820 (17)	−0.0262 (11)	0.0497 (14)	−0.0305 (12)

### Geometric parameters (Å, º)

**Table:**

O1—C8	1.203 (2)	C8—C9	1.510 (2)
N1—C9	1.301 (2)	C10—C11	1.405 (2)
N1—C10	1.378 (2)	C10—C15	1.418 (3)
N2—C1	1.306 (2)	C11—H11	0.9300
N2—C15	1.377 (2)	C11—C12	1.374 (3)
C1—C2	1.471 (2)	C12—C13	1.418 (3)
C1—C9	1.427 (2)	C12—C17	1.505 (3)
C2—C3	1.378 (3)	C13—C14	1.372 (3)
C2—C7	1.401 (2)	C13—C16	1.509 (3)
C3—H3	0.9300	C14—H14	0.9300
C3—C4	1.389 (3)	C14—C15	1.410 (2)
C4—H4	0.9300	C16—H16A	0.9600
C4—C5	1.377 (3)	C16—H16B	0.9600
C5—H5	0.9300	C16—H16C	0.9600
C5—C6	1.385 (3)	C17—H17A	0.9600
C6—H6	0.9300	C17—H17B	0.9600
C6—C7	1.382 (2)	C17—H17C	0.9600
C7—C8	1.491 (2)		
			
C9—N1—C10	114.01 (15)	N1—C10—C15	121.59 (16)
C1—N2—C15	114.02 (15)	C11—C10—C15	119.23 (16)
N2—C1—C2	128.14 (16)	C10—C11—H11	119.1
N2—C1—C9	123.29 (16)	C12—C11—C10	121.88 (19)
C9—C1—C2	108.57 (14)	C12—C11—H11	119.1
C3—C2—C1	130.93 (17)	C11—C12—C13	118.99 (18)
C3—C2—C7	120.44 (17)	C11—C12—C17	119.4 (2)
C7—C2—C1	108.62 (15)	C13—C12—C17	121.62 (19)
C2—C3—H3	120.9	C12—C13—C16	120.3 (2)
C2—C3—C4	118.18 (19)	C14—C13—C12	119.94 (17)
C4—C3—H3	120.9	C14—C13—C16	119.7 (2)
C3—C4—H4	119.4	C13—C14—H14	119.1
C5—C4—C3	121.26 (19)	C13—C14—C15	121.82 (19)
C5—C4—H4	119.4	C15—C14—H14	119.1
C4—C5—H5	119.5	N2—C15—C10	122.55 (15)
C4—C5—C6	121.09 (19)	N2—C15—C14	119.32 (17)
C6—C5—H5	119.5	C14—C15—C10	118.13 (17)
C5—C6—H6	121.0	C13—C16—H16A	109.5
C7—C6—C5	117.92 (18)	C13—C16—H16B	109.5
C7—C6—H6	121.0	C13—C16—H16C	109.5
C2—C7—C8	110.02 (15)	H16A—C16—H16B	109.5
C6—C7—C2	121.10 (17)	H16A—C16—H16C	109.5
C6—C7—C8	128.89 (17)	H16B—C16—H16C	109.5
O1—C8—C7	127.95 (16)	C12—C17—H17A	109.5
O1—C8—C9	127.57 (17)	C12—C17—H17B	109.5
C7—C8—C9	104.47 (14)	C12—C17—H17C	109.5
N1—C9—C1	124.53 (15)	H17A—C17—H17B	109.5
N1—C9—C8	127.17 (15)	H17A—C17—H17C	109.5
C1—C9—C8	108.30 (14)	H17B—C17—H17C	109.5
N1—C10—C11	119.18 (16)		
			
O1—C8—C9—N1	2.1 (3)	C6—C7—C8—C9	178.41 (18)
O1—C8—C9—C1	−178.2 (2)	C7—C2—C3—C4	−1.4 (3)
N1—C10—C11—C12	−179.08 (17)	C7—C8—C9—N1	−179.01 (17)
N1—C10—C15—N2	0.1 (3)	C7—C8—C9—C1	0.66 (18)
N1—C10—C15—C14	179.67 (16)	C9—N1—C10—C11	179.60 (16)
N2—C1—C2—C3	−0.6 (3)	C9—N1—C10—C15	−0.4 (2)
N2—C1—C2—C7	178.74 (17)	C9—C1—C2—C3	179.88 (18)
N2—C1—C9—N1	0.2 (3)	C9—C1—C2—C7	−0.79 (19)
N2—C1—C9—C8	−179.51 (16)	C10—N1—C9—C1	0.3 (2)
C1—N2—C15—C10	0.3 (2)	C10—N1—C9—C8	179.93 (16)
C1—N2—C15—C14	−179.21 (16)	C10—C11—C12—C13	−0.4 (3)
C1—C2—C3—C4	177.84 (18)	C10—C11—C12—C17	179.93 (17)
C1—C2—C7—C6	−178.38 (16)	C11—C10—C15—N2	−179.92 (16)
C1—C2—C7—C8	1.22 (19)	C11—C10—C15—C14	−0.4 (2)
C2—C1—C9—N1	179.72 (16)	C11—C12—C13—C14	−0.7 (3)
C2—C1—C9—C8	0.04 (18)	C11—C12—C13—C16	179.44 (19)
C2—C3—C4—C5	0.9 (3)	C12—C13—C14—C15	1.3 (3)
C2—C7—C8—O1	177.7 (2)	C13—C14—C15—N2	178.80 (17)
C2—C7—C8—C9	−1.16 (19)	C13—C14—C15—C10	−0.8 (3)
C3—C2—C7—C6	1.0 (3)	C15—N2—C1—C2	−179.95 (15)
C3—C2—C7—C8	−179.36 (16)	C15—N2—C1—C9	−0.5 (2)
C3—C4—C5—C6	0.0 (3)	C15—C10—C11—C12	1.0 (3)
C4—C5—C6—C7	−0.5 (3)	C16—C13—C14—C15	−178.83 (19)
C5—C6—C7—C2	−0.1 (3)	C17—C12—C13—C14	178.92 (18)
C5—C6—C7—C8	−179.59 (18)	C17—C12—C13—C16	−0.9 (3)
C6—C7—C8—O1	−2.7 (3)		
